# Effects of Combined Treatment with Ionizing Radiation and the PARP Inhibitor Olaparib in BRCA Mutant and Wild Type Patient-Derived Pancreatic Cancer Xenografts

**DOI:** 10.1371/journal.pone.0167272

**Published:** 2016-12-29

**Authors:** Ines Lohse, Ramya Kumareswaran, Pinjiang Cao, Bethany Pitcher, Steven Gallinger, Robert G. Bristow, David W. Hedley

**Affiliations:** 1 Ontario Cancer Institute and Campbell Family Cancer Research Institute, Princess Margaret Cancer Centre, University Health Network, Toronto, Ontario, Canada; 2 Mount Sinai Hospital, Joseph and Wolf Lebovic Health Complex, Toronto, Ontario, Canada; 3 Translational Research Initiative in Pancreas Cancer, Ontario Institute for Cancer Research, Toronto, Ontario, Canada; 4 Department of Laboratory Medicine and Pathobiology, University of Toronto, Toronto, Ontario, Canada; 5 Department of Medical Oncology and Haematology, Princess Margaret Cancer Center, Toronto, Ontario, Canada; Meharry Medical College, UNITED STATES

## Abstract

**Background:**

The BRCA2 gene product plays an important role in DNA double strand break repair. Therefore, we asked whether radiation sensitivity of pancreatic cancers developing in individuals with germline BRCA2 mutations can be enhanced by agents that inhibit poly (ADP-ribose) polymerase (PARP).

**Methods:**

We compared the sensitivity of two patient-derived pancreatic cancer xenografts, expressing a truncated or wild type BRCA 2, to ionizing radiation alone or in combination with olaparib (AZD-2281). Animals were treated with either a single dose of 12Gy, 7 days of olaparib or 7 days of olaparib followed by a single dose of 12Gy. Response was assessed by tumour growth delay and the activation of damage response pathways.

**Results:**

The BRCA2 mutated and wild type tumours showed similar radiation sensitivity, and treatment with olaparib did not further sensitize either model when compared to IR alone.

**Conclusions:**

While PARP inhibition has been shown to be effective in BRCA-mutated breast and ovarian cancers, it is less well established in pancreatic cancer patients. Our results show no radiosensitization in a germline BRCA 2 mutant and suggest that combining PARP inhibition and IR may not be beneficial in BRCA 2 related pancreatic tumors.

## Introduction

Radiation therapy (RT) plays an important role in the treatment of locally advanced pancreatic cancers, but its effect is limited by the sensitivity of adjacent normal tissues, and the innate radioresistance of these cancers (PMID:24462333). Exposure to ionizing radiation (IR) induces a variety of DNA lesions, of which DNA double strand breaks (DSB) are the most lethal, and, if left unrepaired, lead to genomic instability or cell death. The repair of DSB can be accomplished by two distinct DNA damage repair pathways: non-homologous end joining (NHEJ) and homologous recombination (HR) [1, 2]. Homologous recombination faithfully restores the DNA sequence by using the sister chromatid as a template, and its activity is therefore restricted to the late S and G2 phases of the cell cycle where the sister chromatid is present. It repairs multiple types of DNA damage, including single stranded DNA (ssDNA), DSBs and DNA cross-links [1, 2]. Mutations in proteins essential for HR, such as the breast cancer early onset (BRCA1 & BRCA2) tumor suppressor genes, have been associated with increased risk of tumor development and enhanced sensitivity to chemotherapeutic agents [3, 4, 5, 6, 7].

Non-homologous end joining, which is active during all cell cycle phases, is the main DSB-repair mechanism activated in response to exposure to IR. This pathway catalysis a simple rejoining of two DNA DSB ends without guidance from a template and, as a result, is an error-prone process.

PARP1 is a member of the poly-ADP-ribose polymerase family, a group of enzymes that has been shown to be involved in many processes including DNA repair and cell death [8, 9]. PARP1 plays an important role in the sensing and initiation of DNA repair and been demonstrated to play a role in most forms of DNA repair, including single strand break (SSB) and DSB repair [9, 10, 11]. PARP1 is involved mainly in the repair of single-stranded breaks, which, if unrepaired are converted to DSBs during DNA replication. The mechanisms by which PARP-1 contributes to HR and NHEJ are not as well defined as the role in base excision repair [10, 11].

Because of the essential role of PARP in DSB recognition and repair, PARP inhibitors might sensitize HR defective tumors following exogenous DSBs induced during treatment with IR, resulting in DNA DSB accumulation and cell death.

Consistent with previous observations in a number of solid cancers [12–15], we observed that pre-treatment with the PARP inhibitor olaparib significantly increased the radiosensitivity of genetically engineered mouse breast tumors (Borst and Bristow, manuscript in preparation). Therefore we tested the efficacy of the clinical PARP inhibitor olaparib (AZD-2281) to sensitize a recently-described pancreatic cancer patient-derived xenograft to ionizing radiation.

## Material and Methods

### Primary patient-derived xenografts

Subcutaneous tumors of two primary xenografts, designated as Ontario Cancer Institute Pancreas (OCIP) 23 and 28 were established from pancreatectomy samples superfluous to patient diagnosis using a protocol approved by the University Health Network Research Ethics Board as described previously [3, 16, 17]. Informed consent was obtained from all participating patients. Briefly, tumor fragments were implanted subcutaneously into the flanks of 4–5 week old severe combined immune-deficient mice (SCID). All models used in this study showed first-generation growth and 100% take rate from the third passage on and can be regrown from cryopreserved tumor fragments. The xenograft models closely resemble the morphology of the patient specimen [16, 17] and show stable growth rates over multiple passages.

Patient OCIP28 (http://www.ncbi.nlm.nih.gov/clinvar/RCV000044800) has a clinically-relevant, deleterious germline mutation in BRCA2 that has been described previously [3]. The presence of the patient mutation was confirmed in the xenograft by Sanger sequencing. This xenograft is highly sensitive to cisplatin and gemcitabine, whereas the wild type BRCA control model, OCIP23 is resistant to both agents. These models are otherwise matched in terms of in vivo growth characteristics and tumor hypoxia. Animal experiments were carried out using protocols approved by the University Health Network (UHN) Animal Care Committee under the guidelines of the Canadian Council on Animal Care.

### Treatments

**Fig 1:**
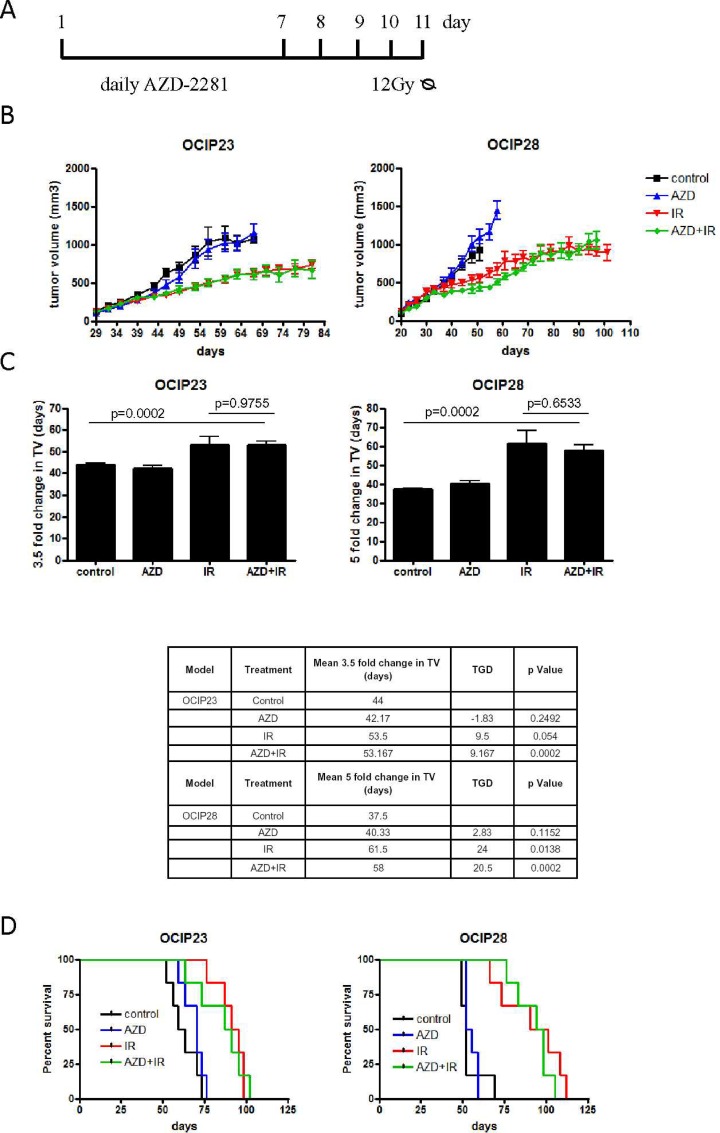
The combination of AZD-2281 and IR did not reduce tumor growth. (A) Tumor bearing animals (n = 10 per group) were treated with vehicle, 7 days of daily 150mg/kg Olaparib po, a single dose 12Gy or the combination of Olaparib and IR. For the combination, animals were treated daily with 150mg/kg Olaparib po for 7 days followed by two days of wash-out and a single dose of 12Gy of IR. Four tumors per group were harvested for histological analysis on day 10. (B) Tumor volume was evaluated 3 times a week. (C) Data from (B) quantitated as fold change in tumor volume (TV) and tumor growth delay (TGD). Error bars represent SD. (D) Survival in response to treatment was measured until mice either reached the humane tumor endpoint or their natural life span in the case of no recurrence of the tumor after treatment.

Olaparib was obtained from UHN Shanghai R&D (Shanghai, PR China). Treatments were started when tumor volume reached approximately 150mm^3^. Animals (n = 10 per treatment group) were treated for 7 days with 150mg/kg olaparib by oral gavage (po), a single dose of 12Gy or both according to the schedule shown in Fig 1A. Animals were irradiated using an X-RAD 225Cx small animal X-Ray irradiator at 225 kVp, 13 mA setting (dose rate of 3.37 Gy/min) with a 1 cm or 1.5 cm collimator and 0.3 mm Cu filter [18]. Four animals per group were sacrificed at 24 hours following radiation treatment. The remaining animals were sacrificed when tumors reached humane endpoint according to institutional guidelines.

Subcutaneous tumors were measured using callipers, and volume calculated according to the formula width^2^×length×0.5. Mean tumor growth delay (TGD) is defined as the difference in time (days) for treated animals to develop tumors that are 3.5 times or 5 times the initial tumor volume for OCIP23 and OCIP28, respectively, compared with animals treated with vehicle control. TGD was calculated by subtracting mean time to change in tumor volume for control animals from mean time to change in tumor volume of the various treatment groups.

### Histological analysis

Tumors were excised, fixed and paraffin embedded. Paraffin tissue sections of 4μm thickness were cut, dried and dewaxed. KU70 staining was performed according to the manufacture’s guidelines using BenchMark XT-an automated slide strainer (Ventana Medical System, Tucson, AZ, USA) with standard antigen retrieval (CC1,Tris/Borate/EDTA pH8.0, #950–124) followed by incubation with the anti- KU70 antibody (1:400) (Kamiya Biomedical Company, MC-351) for 60 minutes. The Ventana Ultraview Universal DAB Detection Kit (#760–500) was used for secondary antibody incubations and slides were counterstained with Bill modified hematoxylin.

For γH2AX staining, endogenous peroxidase was blocked and slides were incubated in γH2AX mouse monoclonal antibody (Millipore, 05–636, 1:1000). Biotinylated anti-mouse IgG incubations were carried out followed by streptavidin biotin detection system (Signet Pathology System, Dedham, MA, USA) for 30 minutes each. Immunoreactivities were revealed by incubation in Nova Red substrate (Vector Lab, Detroit, MI, USA) for 5 minutes and slides were counterstained in Gill modified hematoxylin.

DNAPK and XRCC4 staining was performed according to the manufacture’s guidelines using BenchMark XT-an automated slide strainer with standard antigen retrival (CC1,Tris/Borate/EDTA pH8.0, #950–124). Slides were incubated in DNAPK antibody (Abcam, Cambridge, UK, ab124918, 1: 300) for 32 minutes or XRCC4 antibody (AbD Serotec, Raleigh, NC, USA, AHP387, 1:2000) for 60 minutes. Ventana Ultraview Universal DAB Detection Kit (#760–500) was utilized for detection and slides were counterstained with Bill modified hematoxylin.

Sections were scanned at 20× resolution using an Aperio Scanscope XT scanner (Aperio Technologies, Vista, CA, USA). Images were analyzed using the Aperio ImageScope software ver. 11.1.2.752, positive pixel count algorithm (PPC). Necrotic areas were excluded from the analysis.

### Statistical analysis

Tumor growth data were analyzed using mixed effect modeling which accounts for correlations among the measurements of the same model. To determine whether the effect of treatment on growth rate differed between OCIP23 and OCIP28, another mixed effects model that included interactions between treatment, xenograft model and time was used. The significance of interaction terms was determined using Wald tests. The survival percentages for the in vivo data were calculated using the Kaplan-Meier technique and the curves were tested for significance using the log-rank test. The Kruskal-Wallis test was used to determine if treatment was associated with the percent of DNAPK, Ku70, XRCC4 and γH2AX. The Mann-Whitney test was employed to make pairwise comparisons between the four treatment groups.

## Results

### BRCA2 mutated pancreatic tumors are not always sensitized to ionizing radiation

To test the radiosensitivity of the BRCA2 mutant (mt) and BRCA wt xenografts, tumor-bearing animals were treated with a single dose of 12Gy. Significant growth inhibition was seen in both models (OCIP23 p<0.0001; OCIP28 p = 0.0075) compared with untreated control mice (Fig 1B), and survival was significantly increased (OCIP23 p = 0.00051; OCIP28 p = 0.002) (Fig 1C). Although the radiation sensitivity of the two models was similar based on tumor growth inhibition, survival was significantly increased in OCIP28 compared to OCIP23 (p = 0.046; 29 days in OCIP23 versus 38days in OCIP28) (Fig 1C).

### Effects of olaparib and in combination with ionizing radiation

To investigate the potential efficacy of the combination of olaparib and IR, tumor bearing mice were treated with 150mg/kg olaparib, 12Gy of IR or the combination of olaparib and IR according to the schedule shown in Fig 1A. For the combination treatment, animals were treated with olaparib for 7days followed by two days of drug wash-out followed by a single dose of 12Gy of irradiation.

**Fig 2:**
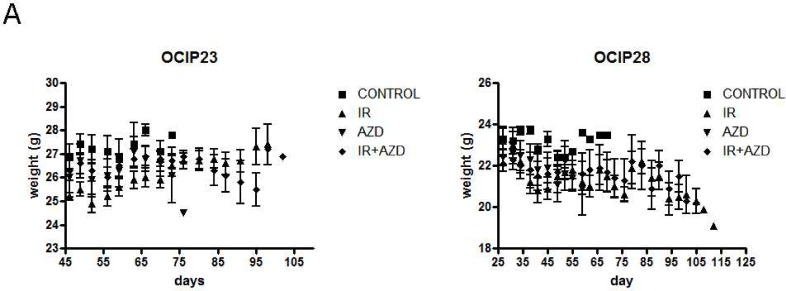
Treatment with the combination of AZD-2281 and IR did not impact tumor weight. Tumor weight was evaluated three times a week over the course of the experiment. No significant weight loss was observed in any of the treatment groups.

No loss in body weight was observed in response to treatment (Fig 2).

Treatment with olaparib alone did not affect tumor growth in either xenograft model (OCIP23 p = 0.8; OCIP28 p = 0.61) when compared to untreated controls (Fig 1B and 1C). While IR alone significantly reduced tumor growth, pre-treatment with AZD-2281 did not further reduce tumor growth (OCIP23 p = 0.89; OCIP28 p = 0.97) in either model (Fig 1B and 1C). We also noted that the combination of AZD-2281 and IR did not increase survival when compared to untreated control tumors or tumors treated with IR, respectively (OCIP23 p = 0.77; OCIP28 p = 0.45) (Fig 1D).

### Analysis of NHEJ repair activation in response to treatment with AZD-2281 and IR

**Fig 3:**
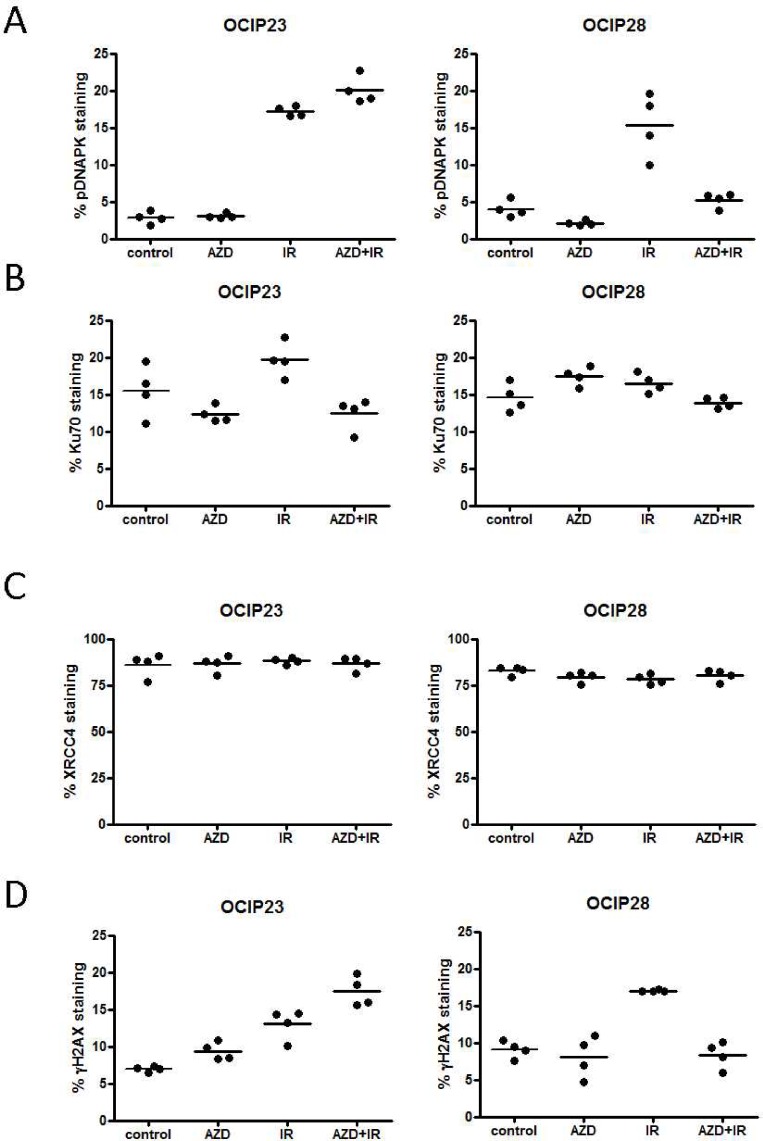
Treatment of the BRCA wt tumor OCIP23 results in accumulation of γH2AX positive nuclei. FFPE sections (n = 4 per group) were stained for (A) DNAPK, (B) Ku70, (C) XRCC4 or (D) γH2AX and analyzed using the Aperio ImageScope software positive pixel count algor.

**Fig 4:**
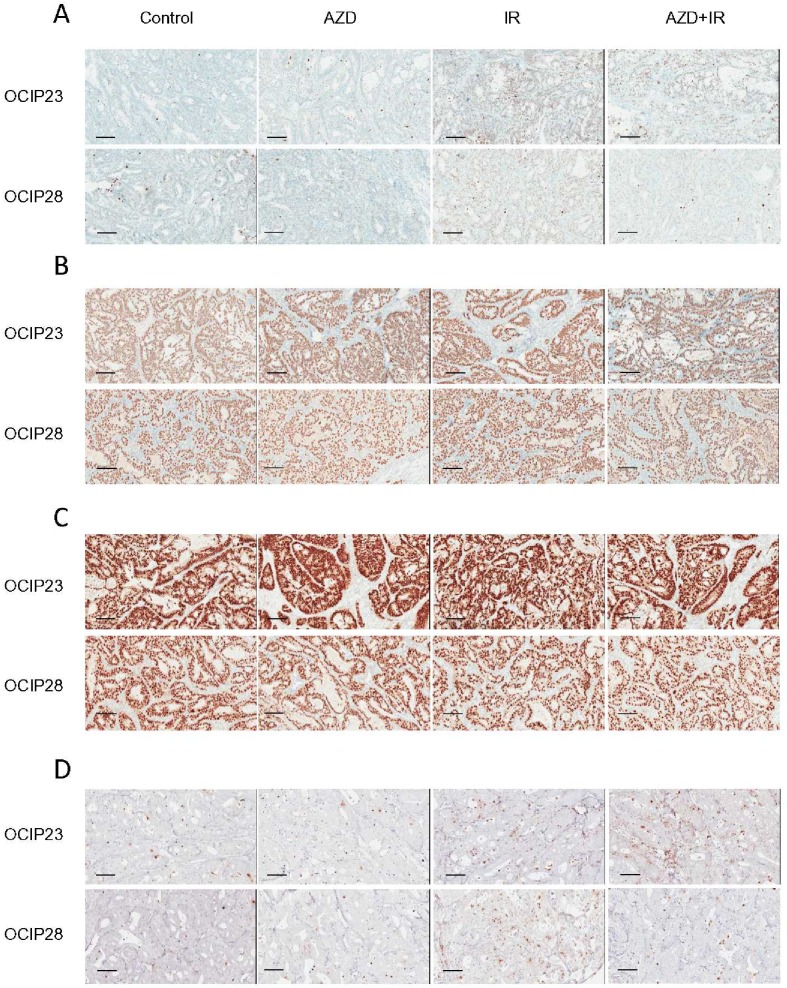
Treatment of the BRCA wt tumor OCIP23 results in accumulation of γH2AX positive nuclei. Representative images of FFPE sections (n = 4 per group) stained for (A) DNAPK, (B) Ku70, (C) XRCC4 or (D) γH2AX. Bars represent 100μm.

We have previously demonstrated that the BRCA2 mt OCIP28 shows aberrant Rad51 foci [3]. Therefore we analysed the expression of three key proteins involved in NHEJ: DNAPK, Ku70 and XRCC4 following treatment. No difference in DNAPK phosphorylation or Ku70 and XRCC4 expression was observed between the BRCA2 wt model OCIP23 and BRCA2 mt model OCIP28 (Fig 3A, 3B and 3C). DNAPK phosphorylation was significantly increased (p = 0.029) in the BRCA2 wt model OCIP23 in response to IR compared to the untreated control (Figs 3A and 4A). While treatment with olaparib alone did not impact DNAPK phosphorylation, the combination with IR significantly increased (p = 0.029) DNAPK phosphorylation when compared to IR alone (Fig 3A). This was not observed in the BRCA2 mt OCIP28. Indeed, treatment with AZD-2281 reduced DNAPK phosphorylation (p = 0.029) when compared to untreated control tumors. While treatment with IR alone increased DNAPK phosphorylation (p = 0.029) when compared to control tumors, DNAPK phosphorylation was significantly reduced in response to the combination of AZD-2281 and IR (p = 0.029). The DNAPK phosphorylation of tumors treated with the combination was comparable to untreated controls (p = 0.2) (Fig 3A).

Ku70 expression in OCIP23 was not impacted by treatment with either olaparib (p = 0.34) or IR (p = 0.081) when compared to untreated control tumors. However, the combination treatment significantly reduced Ku70 expression (p = 0.02) compared to irradiation alone (Figs 3B and 4B). Similar to OCIP23, treatment of OCIP28 with olaparib (p = 0.057) or IR (p = 0.34) alone did not increase expression of Ku70, while the combination resulted in reduced Ku70 expression (p = 0.029; Fig 2B). No differences in XRCC4 expression were observed in response to either treatment (Figs 3C and 4C).

### The combination of IR and AZD-2281 results in reduced DNA damage in BRCA mutant xenografts

Previous studies have suggested that treatment with PARP inhibitors in combination with DNA damaging agents such as cisplatin or IR can result in the accumulation of DSB and γH2AX foci [19, 20]. In the BRCA wild type model OCIP23, treatment with both olaparib and IR as single agents resulted in the accumulation of γH2AX foci (AZD-2281 p = 0.029, IR p = 0.029) when compared to untreated control tumors. Treatment with the combination of AZD-2281 and IR resulted in a further increase in γH2AX accumulation when compared to treatment with IR alone in OCIP23 xenografts ((p = 0.029), Figs 3D and 4D). However, this was not the case with the BRCA2 mt OCIP28. Here, although treatment with IR led to a significant accumulation of γH2AX foci (p = 0.029), this effect was not seen with olaparib given as a single agent (p = 0.89), and in contrast to OCIP23 the combination of AZD-2281 and IR resulted in a reduction of γH2AX foci (p = 0.029) when compared to IR alone (Fig 3D).

## Discussion

We have recently shown that germline mutations of BRCA1 and BRCA2 sensitize pancreatic cancers to treatment with cisplatin and gemcitabine [3]. Since treatment with these two agents gives rise to different types of DNA lesions, resulting in DSBs of varying complexity [19, 20], these results suggested that pancreatic cancers with BRCA mutations show a more generalised sensitivity to DNA damaging agents, rather than sensitivity to a specific type of lesion. However, no significant increase in radiosensitivity was observed when one of these germline BRCA-mutant models was compared to a BRCA wild type control. While treatment with both cisplatin and gemcitabine induces complex DNA lesions that require repair through the HR pathway [21], the majority of lesions induced by IR can be repaired through NHEJ which does not depend on BRCA2 activity [22]. This might explain the difference between chemo- and radiosensitivity in the HR deficient model.

PARP is an important protein in DNA repair pathways especially in BER where PARP is involved in repair of SSBs. While PARP inhibition is functionally silent in HR proficient cells, PARP inhibition leads to the accumulation of SSBs and the development of DSBs in cells deficient in HR [19, 20]. A number of preclinical studies in a variety of cancer types, such as breast, ovarian and prostate cancer, have shown that PARP inhibition can enhance the effects of various chemotherapies and IR [12–15, 18, 20, 23, 24, 25]. In pancreatic cancer, however, responses to treatment with PARP inhibitors have been less impressive [26].

In addition to its function in BER, PARP has also been shown to be involved in the regulation of DSB repair pathways [17, 18, 27]. PARP has been shown to regulate repair through the error-prone NHEJ pathway by inhibiting DNAPK activation [27]. In HR deficient cells, PARP inhibition results in NHEJ activation leading to accelerated DSB repair, increased mutations, chromosomal rearrangements and eventually NHEJ-mediated cell death [27].

In order to investigate whether the radiosensitivity of HR deficient tumors can be increased with the use of PARP inhibitors, we further investigated the efficacy of olaparib in combination with IR. We observed rapid resolution of DNA DSB breaks in the HR deficient patient-derived xenograft model in response to PARP inhibition. This increase in DNA damage repair may be attributed to the dysregulation of NHEJ and activation of DNAPK, resulting in low levels of persistent DSBs and rapid dephosphorylation of DNAPK that was not observed in the HR proficient xenograft model. In contrast to results shown in prostate cancer [18], no radiosensitization was observed in the BRCA2 germline mutant tumor in response to treatment with AZD-2281. This is consistent with previous observations made by Karnak et al., where treatment with olaparib sensitized pancreatic cancer cells lines to IR *in vitro* but failed to reduce tumor growth *in vivo* [26].

Early observations suggest that pancreatic tumors show unusually high levels of chromothriptic events resulting in structural rearrangements, switches in copy number state and retention of heterozygosity [28–30]. Exposure to multiple chromothriptic events early in pancreatic tumor development might lead to the development of yet unexplained coping mechanisms and allow tumor cell survival despite increased mutation rates resulting from NHEJ repair. This hypothesis might provide an explanation for treatment resistance observed in this study.

While recent studies have increased our knowledge of the regulation of DNA DSB repair mechanisms and PARP function, further studies are needed to fully understand the complex interactions between different pathways and the survival strategies that are “hitchhiked” by pancreatic cancer cells to evade persistent DNA damage and cell death.
